# Instructing Students in the Home Health Setting: An Innovative Interprofessional Education Experience

**DOI:** 10.1007/s40670-025-02506-5

**Published:** 2025-09-04

**Authors:** Stacia Hall Thompson, Charlotte Navarro

**Affiliations:** 1 Physical Therapy Department, Methodist University, Fayetteville, NC USA; 2 Occupational Therapy Department, Methodist University, Fayetteville, NC USA

**Keywords:** Interprofessional education, Home health setting, Simulation

## Abstract

Limited information exists on effective instruction in interprofessional practice in the home health setting (HHS) for allied health students. Intentionally designed simulated interprofessional education experiences (SIPE) may prepare students for real-world scenarios. This innovative paper focuses on discovering if a SIPE in the HHS is a positive learning experience.

Nurses, Occupational Therapists, and Physical Therapists practice in many settings, including patients’ homes. A simulated interprofessional education (SIPE) activity is one way to instruct students on the intricacies associated with the home health setting (HHS). Guided by Kolb’s Experiential Learning Theory, SIPE is a pedagogical teaching strategy that provides students with opportunities to learn through concrete experiences, reflection, conceptualization, and experimentation [1]. Following Kolb’s principles, an HHS SIPE provides students with an opportunity to engage in a simulated event and receive feedback during a debrief, allowing them to connect classroom knowledge with clinical practice, fostering authentic, realistic, and meaningful learning in a low-risk, safe, and controlled environment [1, 2].

Current evidence demonstrates that students value virtual and apartment-based HHS SIPE activities in didactics to improve communication skills and understand discipline-specific roles [1, 2]. However, limited evidence focuses on individual aspects of the HHS, such as recognizing safety concerns or understanding the effectiveness of the structure of an HHS SIPE. Therefore, this innovation paper discusses an HHS SIPE activity that concentrates on patient safety, assessing student perceptions of the effectiveness of the SIPE in improving learning outcomes.

Figure 1 illustrates the HHS safety SIPE activity, in which 65 students (18 undergraduate nursing and 47 OT and PT graduate students) participated. Two nursing, one OT, and three PT faculty members guided the SIPE. To demonstrate their understanding of the SIPE objectives, students complete a pre-briefing, participate in the SIPE activity, and engage in a debriefing by sharing knowledge gained.

**Fig. 1:**
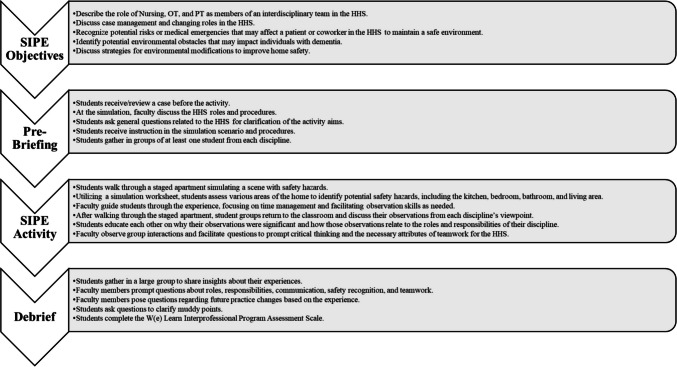
Home health setting simulated interprofessional experience activity

To guide the experience, students follow a written clinical case involving an older adult with dementia and a history of falls. Before entering the SIPE activity, students identify problems related to home safety based on the clinical case. In collaborative teams that include all disciplines, students enter the SIPE activity, observing a staged apartment simulating an unsafe environment. A faculty member cues students to notice items impacting home safety, such as a knife left out in the kitchen or clothes lying on the floor in the bedroom. After observing all rooms, student groups exit the activity, reflect on the experience, and connect the identified safety concerns from the written clinical case to the unsafe home environment. Students share their expertise regarding discipline-specific roles and responsibilities to determine how to address each identified safety concern. During the debriefing, faculty provide guiding questions to facilitate the sharing of newly learned information.

After completing the experience, students completed the W(e) Learn Interprofessional Program Assessment Scale (WLIPAS) [3]. This scale gathers the student's perspective on the *structure, content, service*, and *outcomes* of the activity [3]. The *structure* comprises teaching and facilitation strategies, while *content* encompasses authenticity and responsiveness to student needs. *Service* encompasses assistance from faculty, the organization, and provided resources. *Outcomes* include satisfaction, knowledge improvement, and attitude change [3]. Students rate their level of agreement with 30 statements on a seven-point Likert scale ranging from one (strongly disagree) to seven (strongly agree) [3].

Data from 65 students averaged a score of 6.60 (SD = 0.131) out of seven for all 30 questions, indicating that students agreed and strongly agreed that the activity was beneficial in all areas. The highest-rated question (M = 6.79, SD = 0.477) related to applying knowledge to future practice. Students rated the provision of resources as the lowest (M = 6.32, SD = 1.00). Table 1 lists discipline-specific average results.

**Table 1:** Results

Average of Results n = 65	Nursing Average n = 18	Occupational Therapy Average n = 27	Physical Therapy Average n = 20
6.60 (SD = 0.131)	6.68 (SD = 0.095)	6.64 (SD = 0.192)	6.51 (SD = 0.156)

*Average of 30 questions

Students positively perceived the HHS safety SIPE, as evidenced by high average scores in all content areas on the WLIPAS. Based on the WLIPAS domains, the data suggest that the HHS SIPE creates an authentic experience that produces positive outcomes in learning. Despite evidence that virtual HHS SIPE is feasible and supports student learning [1], the use of an in-person apartment-based approach may be a pedagogical advantage because it promotes realistic scenarios and interactions among students simulating a real-life situation [2]. As provision of resources was rated lowest, future HHS SIPE may benefit from additional resources, such as an introductory podcast or presentation on general requirements for working in the HHS, to help guide learning [1].

A limitation of this study is the lack of qualitative inquiry and thematic analysis to explain student perceptions and triangulate results. Furthermore, this activity exposed students to the safety domain in HHS. Other domains, such as emergency medical assessment, could provide an enhanced learning experience. The HHS SIPE is a positively perceived learning activity that offers an opportunity for authentic learning that mirrors clinical practice, which faculty could embed in didactic training to prepare students for future clinical practice.
